# Supplementary material for: A novel Vector-Symbolic Architecture for graph encoding and its application to viral pangenome-based species classification
Source: BioData Min. 2026 May 17;19:54. doi: 10.1186/s13040-026-00561-1 (PMC13361823; doi:10.1186/s13040-026-00561-1)
Supplement: Supplementary file 1 — Supplementary Material 1 [file 13040_2026_561_MOESM1_ESM.pdf]

A novel Vector-Symbolic Architecture for graph encoding and its application to viral pangenome-based species classification

Fabio Cumbo[1] (0000-0003-2920-5838), Kabir Dhillon[2] (0009-0000-3830-1405), Jayadev Joshi[1] (0000-0001-7589-5230), Davide Chicco[3,4] (0000-0001-9655-7142), Sercan Aygun[5] (0000-0002-4615-7914), and Daniel Blankenberg[1,6,*] (0000-0002-6833-9049)

[1] Computational Life Sciences, Cleveland Clinic Research, Cleveland Clinic, Cleveland, OH 44195, USA
[2] College of Engineering, Ohio State University, Columbus, OH 43210, USA
[3] Dipartimento di Informatica Sistemistica e Comunicazione, Università di Milano-Bicocca, Milan, 20125 MI, Italy
[4] Institute of Health Policy Management and Evaluation, University of Toronto, Toronto, ON M5T 3M6, Canada
[5] School of Computing and Informatics, University of Louisiana at Lafayette, Lafayette, LA 70504, USA
[6] Department of Molecular Medicine, Cleveland Clinic Lerner College of Medicine, Case Western Reserve University, Cleveland, OH 44195, USA

* To whom correspondence should be addressed:
Daniel Blankenberg[1,6], Computational Life Sciences, Cleveland Clinic Research, Cleveland Clinic, 9500 Euclid Avenue, NA2, Cleveland, OH 44195, USA.
Email: blanked2@ccf.org

Cross-disciplinary terminology standardization

Because our classification framework integrates concepts from genomics, graph theory, and Hyperdimensional Computing (Vector-Symbolic Architectures), the underlying terminology naturally spans multiple scientific domains. To ensure absolute clarity for readers from diverse computational and biological backgrounds, we have systematically standardized our terminology throughout the main manuscript.

The table below provides a direct mapping of the core conceptual roles to their respective biological, graph-theoretic, and VSA-specific terms, culminating in the finalized standardized terminology adopted in this study. This table served as a quick-reference guide to clarify how distinct domain concepts (e.g., biological species, graph node weights, and VSA hypervectors) converge into single mathematical and structural representations within our pipeline.

**Conceptual Role**	**Biological Term**	**Graph Representation**	**VSA-Model Term**	**Standardized Term (Main Text)**
**Class Identity**	Taxonomic label (species/genus)	Node label / class weight	Species hypervector	**Species hypervector**

**Class Representation**	Species	Node / class representation	Prototype hypervector	**Species hypervector**
**Structural Relation**	Adjacency / co-occurrence	Edge	Bound hypervector	**Edge** (reconstructed)
**Structure Validation**	Edge existence	Edge detection / recovery	Matching in HV space	**Edge reconstruction**
**Model Consistency**	-	-	Reconstruction score	**Reconstruction rate**
**Model Interpretation**	-	-	Confidence score	**Internal coherence** (via reconstruction rate)

Table S1: Cross-disciplinary terminology mapping and standardization. This table provides a unified lexicon to bridge the distinct scientific domains integrated within the classification framework: viral taxonomy (Biological Term), topological data structures (Graph Representation), and hyperdimensional computing (VSA-Model Term). Domain-specific terminology sharing the same conceptual role has been systematically consolidated.

Advantages of Hyperdimensional Computing (HDC)

- Robustness to noise and error: HDC uses high-dimensional vectors, distributing information across all dimensions. Scalars or symbols are represented using long-dimensional vectors, where the cumulative counts of binary values and their ratios encode the information. However, this encoding is fundamentally different from traditional binary radix-2 representation, as it does not assign significance to any specific bit. This inherent redundancy makes HDC highly tolerant to noise and errors, unlike conventional computing, where a single bit flip (e.g., the most significant or sign bit) can drastically alter the outcome. This robustness is crucial for real-world applications where data can be noisy or incomplete [1,2];
- Computational efficiency: HDC operations are simple and highly parallelizable, leading to fast computation, especially for tasks like similarity search in high-dimensional spaces. Algebraic operations (e.g., multiplication and addition) can be implemented with simple logic during encoding, which reduces computational complexity, particularly in application-specific hardware designs [3,4];
- One/Few-shot learning: HDC can often learn from single examples, unlike deep learning, which requires extensive training, large datasets, batching, and randomization. In contrast, HDC depends only loosely on repeated data input and requires little to no heavy feature engineering. This ability to generalize from limited data makes it suitable for situations where training data is scarce or expensive [5–7];
- Power and energy efficiency: due to its simple and parallelizable operations, HDC can be implemented in power- and energy-efficient hardware. Recent advances in vector design techniques further improve this efficiency: shorter vectors enable lightweight architectures

with lower latency, making HDC particularly advantageous in energy-constrained scenarios [8,9];

- Interpretability and transparency: the symbolic nature of HDC can make its operations more interpretable than those of deep learning models, which are often considered "black boxes" [10,11];
- Scalability: following the previous point about the computational efficiency, HDC can perfectly scale to large datasets. Through encoding, repeated or complex data can be compressed into one-dimensional vectors, which enables efficient representation and supports scalability across diverse data sizes [12,13];
- Flexibility and generality: HDC can represent and manipulate a wide range of data types and structures [14], making it adaptable to diverse domains and applications [15]. This flexibility is evident in its use for various bioinformatics tasks [16,10] as well as other applications like natural language processing [17], artificial intelligence [18,19], robotics [20], among other scientific domains.

Baseline Comparison with Mash

To contextualize the classification performance of our framework, we conducted an empirical baseline comparison using Mash [21] (version 2.3), a widely adopted MinHash-based alignment-free sequence distance estimator. The benchmarking utilized the exact same data splits (542 training genomes, 542 test genomes) as the primary VSA experiments. Training genomes were sketched using *mash sketch* (default parameter), and test genomes were classified by assigning them to the taxonomic label of the training genome with the minimum Mash distance value.

Under this baseline approach, Mash achieved a species-level accuracy of 82.75%. In comparison, our VSA-based graph framework achieved an accuracy of 87.08%. This result is highly encouraging. A direct performance comparison between these two methods is inherently unbalanced due to their fundamentally different underlying paradigms: Mash utilizes a standard “bag-of-words” approach, directly comparing flat sets of extracted minimizers. In contrast, our VSA approach must mathematically compress and maintain a massive, complex topological data structure, a weighted *de Bruijn* graph, into a single, fixed-dimensional hypervector representation. The VSA model must cope with the signal degradation inherent to mathematical superposition and binding of structural edges, constraints that set-based sketches like Mash completely bypass. Therefore, the fact that our VSA model not only overcomes these immense representational constraints but actually outperforms the established Mash baseline demonstrates that it successfully captures and leverages meaningful biological and topological signals well beyond standard k-mer summaries.

Benchmarking against established viral classification tools

Standard viral classification tools, such as VIRIDIC [22] and vConTACT3 [23], rely fundamentally on all-vs-all pairwise comparison between sequences to compute intergenomic distances or protein-sharing networks. Consequently, the computational complexity of these approaches scales quadratically with the number of input sequences.

While this quadratic scaling makes traditional pairwise-based tools computationally expensive and sometimes prohibitive for massive genomic datasets, our benchmarking was conducted exclusively on our set of 542 genomes (one representative for each of the 542 species) used to test our model. Both the baseline tools and our VSA framework were evaluated on this identical 542-genome subset.

Established tools like VIRIDIC and vConTACT3 inherently generate *de novo* viral clusters rather than assigning genomes to predefined supervised labels. VIRIDIC outputs strict anonymous numeric cluster IDs (e.g. "*Cluster 255*"), making standard supervised classification metrics fundamentally inapplicable. Conversely, vConTACT3 outputs placeholder taxonomic strings (e.g., "*novel_genus_5*"). By mapping these predicted placeholder clusters to their majority ground-truth taxonomic labels, we can accurately compute a mapped accuracy to evaluate its predictive power relative to our framework.

On the test set, mapping vConTACT3's clusters to their majority ground-truth labels yielded an accuracy of 90.96%, compared to the 60.51% accuracy of our VSA flat genus model.

However, to properly evaluate the direct topological agreement between the two distinct methodologies, we evaluate pure structural clustering concordance. It is important to note a mathematical artifact regarding standard partition-agreement metrics like the Adjusted Rand Index (ARI) on this specific test set. Because the dataset contains one representative genome per species, accurate clustering algorithms will naturally isolate almost every genome into its own singleton cluster (e.g., VIRIDIC generated 534 unique clusters for the 542 genomes). In such high fragmented, singleton-dominated partitions, the expected pairwise chance agreement utilized in the ARI formula effectively cancels out the actual agreement, artificially suppressing the adjusted score to near zero.

Therefore, to rigorously evaluate structural concordance without singleton suppression, we utilized the Normalized Mutual Information (NMI) metric, which measures the amount of information shared between the two clustering configurations, scaling the score from 0 (completely independent) to 1 (perfectly identical groupings) regardless of the arbitrary names assigned to the clusters. At the species level, the clustering agreement between our VSA model and VIRIDIC yielded an NMI of 0.98. On the other hand, at the genus level, the topological agreement between the VSA model and vConTACT3 yielded an NMI of 0.85.

Additional Supplementary Tables

Supplementary Table S2

Table S2: List of Accession Numbers of the reference genomes retrieved from NCBI GenBank alongside their quality information and their full taxonomic label as reported by NCBI.

Supplementary Table S3

Table S3: List of Accession Numbers used for testing the VSA-based graph model at the species level with their taxonomic label as provided by NCBI GenBank, their predicted taxonomy, and reconstruction rate.

Supplementary Table S4

Table S4: List of Accession Numbers used for testing the VSA-based graph model at the genus level with their taxonomic label as provided by NCBI GenBank, their predicted taxonomy, and reconstruction rate.

Supplementary Table S5

Table S5: List of Accession Numbers used for testing the VSA-based genus graph models at the species level via hierarchical classification, with their taxonomic label as provided by NCBI GenBank, their predicted taxonomy, and reconstruction rate.

References

1. Zhang S, Wang R, Zhang JJ, Rahimi A, Jiao X. Assessing robustness of hyperdimensional computing against errors in associative memory : (invited paper). 2021 IEEE 32nd International Conference on Application-specific Systems, Architectures and Processors (ASAP). IEEE; 2021. doi:10.1109/asap52443.2021.00039

2. Zhang S, Juretus K, Jiao X. Exploring hyperdimensional computing robustness against hardware errors. IEEE Trans Comput. 2025;74: 1963–1977. doi:10.1109/tc.2025.3547142

3. Kang J, Khaleghi B, Rosing T, Kim Y. OpenHD: A GPU-Powered Framework for Hyperdimensional Computing. IEEE Trans Comput. 2022;71: 2753–2765. doi:10.1109/tc.2022.3179226

4. Salamat S, Imani M, Rosing T. Accelerating hyperdimensional computing on FPGAs by exploiting computational reuse. IEEE Trans Comput. 2020;69: 1159–1171. doi:10.1109/tc.2020.2992662

5. Nair DR, Purushothaman A. Brain Inspired One Shot Learning Method for HD Computing. VLSI Design and Test. 2019; 286–297. doi:10.1007/978-981-32-9767-8_25

6. Burrello A, Schindler K, Benini L, Rahimi A. One-shot learning for iEEG seizure detection using end-to-end binary operations: Local binary patterns with hyperdimensional computing. 2018 IEEE Biomedical Circuits and Systems Conference (BioCAS). IEEE; 2018. doi:10.1109/biocas.2018.8584751

7. Burrello A, Schindler K, Benini L, Rahimi A. Hyperdimensional Computing With Local Binary Patterns: One-Shot Learning of Seizure Onset and Identification of Ictogenic Brain Regions Using Short-Time iEEG Recordings. IEEE Trans Biomed Eng. 2020;67: 601–613. doi:10.1109/TBME.2019.2919137

8. Rahimi A, Kanerva P, Rabaey JM. A Robust and Energy-Efficient Classifier Using Brain-Inspired Hyperdimensional Computing. Proceedings of the 2016 International Symposium on Low Power Electronics and Design. New York, NY, USA: Association for Computing Machinery; 2016. pp. 64–69. doi:10.1145/2934583.2934624

9. Imani M, Huang C, Kong D, Rosing T. Hierarchical hyperdimensional computing for energy efficient classification. Proceedings of the 55th Annual Design Automation Conference. New York, NY, USA: ACM; 2018. doi:10.1145/3195970.3196060

10. Stock M, Van Criekinge W, Boeckaerts D, Taelman S, Van Haeverbeke M, Dewulf P, et al. Hyperdimensional computing: A fast, robust, and interpretable paradigm for biological data. PLoS Comput Biol. 2024;20: e1012426. doi:10.1371/journal.pcbi.1012426

11. Chen H, Ni Y, Huang W, Imani M. Scalable and interpretable brain-inspired hyper-dimensional computing intelligence with hardware-software co-design. 2024 IEEE Custom Integrated Circuits Conference (CICC). IEEE; 2024. doi:10.1109/cicc60959.2024.10529049

12. Thomas A, Khaleghi B, Jha GK, Dasgupta S, Himayat N, Iyer R, et al. Streaming Encoding

Algorithms for Scalable Hyperdimensional Computing. 2022. Available: http://arxiv.org/abs/2209.09868

13. Parikh D, Prasanna V. ScalableHD: Scalable and High-Throughput Hyperdimensional Computing Inference on Multi-Core CPUs. 2025. Available: http://arxiv.org/abs/2506.09282

14. Kleyko D, Rachkovskij DA, Osipov E, Rahimi A. A survey on Hyperdimensional Computing aka Vector Symbolic Architectures, part I: Models and data transformations. ACM Comput Surv. 2023;55: 1–40. doi:10.1145/3538531

15. Kleyko D, Rachkovskij D, Osipov E, Rahimi A. A survey on Hyperdimensional Computing aka Vector Symbolic Architectures, part II: Applications, cognitive models, and challenges. ACM Comput Surv. 2023;55: 1–52. doi:10.1145/3558000

16. Cumbo F, Chicco D. Hyperdimensional computing in biomedical sciences: a brief review. PeerJ Comput Sci. 2025;11: e2885. doi:10.7717/peerj-cs.2885

17. Quiroz-Mercado JI, Barrón-Fernández R, Ramírez-Salinas MA. Semantic Similarity Estimation Using Vector Symbolic Architectures. IEEE Access. 2020;8: 109120–109132. doi:10.1109/ACCESS.2020.300176

18. Evaluating Complex Sparse Representation of Hypervectors for Unsupervised Machine Learning. [cited 29 May 2025]. Available: https://doi.org/10.1109/IJCNN55064.2022.9892981

19. Osipov E, Kahawala S, Haputhanthri D, Kempitiya T, De Silva D, Alahakoon D, et al. Hyperseed: Unsupervised Learning With Vector Symbolic Architectures. IEEE Trans Neural Netw Learn Syst. 2024;35: 6583–6597. doi:10.1109/TNNLS.2022.3211274

20. Neubert P, Schubert S, Protzel P. An Introduction to Hyperdimensional Computing for Robotics. KI - Künstliche Intelligenz. 2019;33: 319–330. doi:10.1007/s13218-019-00623-z

21. Ondov BD, Treangen TJ, Melsted P, Mallonee AB, Bergman NH, Koren S, et al. Mash: fast genome and metagenome distance estimation using MinHash. Genome Biol. 2016;17: 132. doi:10.1186/s13059-016-0997-x

22. Moraru C, Varsani A, Kropinski AM. VIRIDIC—A novel tool to calculate the intergenomic similarities of prokaryote-infecting viruses. Viruses. 2020;12: 1268. doi:10.3390/v12111268

23. Bolduc B, Zablocki O, Turner D, Bin Jang H, Guo J, Adriaenssens EM, Dutilh BE, Sullivan MB. Machine learning enables scalable and systematic hierarchical virus taxonomy. Nature Biotechnology. 2025;19: 1. doi:10.1038/s41587-025-02946-9
